# Proceedings of the Kentucky State Medical Society

**Published:** 1852-11

**Authors:** 


THE WESTERN JOURNAL.

THIRD SERIES.—VOL. X.—NO. V.

LOUISVILLE, NOVEMBER 1, 1852.

### THE KENTUCKY STATE MEDICAL SOCIETY.

The readers of this *Journal* will remember that we gave an account, last year, of the formation of a State Medical Society, in Kentucky. Last year the Society was formed, and, after organizing, and preparing work for a future meeting, it adjourned to meet in Louisville on the 20th October, 1852. The recent meeting, therefore, may be regarded as the first meeting of the Society, and we are sure that we express the feelings of all the members when we say that it is scarcely possible that a more pleasant meeting of the kind ever was held. Every one seemed to be animated by one feeling, and that was devoted to the success) the cause. The physicians of Louisville united themselves in numbers with the Society, and every thing was conducted with the greatest harmony and good will. We do not remember ever to have seen a larger amount of business completed by any Society in the same space of time.

When the Declaration of Independence was announced, it made no change in the actual state of the contest with Great Britain. It announced a position; it proclaimed a resolution, a determination to accomplish a result; but how far that result lay in the future, it did not proclaim nor indicate. But it accomplished some great ends. It made those who were engaged in the lingering and disputed struggle feel that they had a country; that her head was erect among the nations, and that all who were engaged in the cause were many degrees above the character of mere rebels. In a miniature form these must be the feelings of those who witnessed the sessions of the recent meeting of the State Medical Society in this city. The physicians of Kentucky must rejoice to know that they have a rallying point now for professional advancement, and professional honor. All must now feel that the medical men of Kentucky have harnessed themselves for their duties to humanity, to science, and to their profession. These were the feelings that animated us as we looked upon the proceedings of the Society, and they were strengthened by all that we saw. It was an animating scene to look upon those who had attained the highest positions in their profession, as well as those who occupied humbler places, working sturdily together towards a common point, and we felt that the glory, honor and dignity of a profession, which, in the expressive language of the Louisville Chancellor, "is, at least, next to the profession of the legate of the skies," were in hands that would protect them. May the spirit of labor, and devotion to the interests of medical science, that moved the members of the Society at its recent meeting, ever characterize the movements of the association, and thus advance alike the interests of the profession and of society.

As a matter of interest to the profession, we publish a synopsis of the proceedings of the Kentucky State Medical Society, at its recent meeting in this city.

At the second annual meeting of the State Medical Society, held in the Circuit Court Room, in Louisville, on the 20th day of October, 1852, at 1 o'clock, P. M., the President, Dr. Wm. L. Sutton, took the chair, and called the Society to order.

Dr. J. B. Flint offered an amendment to the second section of article three of the Constitutien. The amendment is for the purpose of increasing the facilities for admitting members. It was not acted upon at this session, and lies over for consideration next year.

The roll of members was called, and a considerable proportion o the original members answered to their names.

A large number of new names was offered for membership, and, during the progress of the session, some seventy new members were enrolled.

AFTERNOON SESSION.

The Committee appointed to report on the order of proceedings, reported a series of recommendations. which was adopted. One of the series, which provided for the nomination of the officers of the Society by a committee, was afterwards stricken out.

On motion of Dr. Gross, the thanks of the Society were given to the Indiana State Medical Society for copies of the proceedings of the third annual meeting of that Society, and, upon motion of Dr. L. P. Yandell, Drs. Clapp, Leonard and Steele, of New Albany, Ia., who were present, were invited to take seats with the Society, and participate in the proceedings.

Dr. Bell presented the form of case-book modelled by the President of the Society, which he moved to refer to a committee to report upon. Committee: Drs. Bell, Foree, and Chipley.

On motion of Dr. Raphael, a committee was appointed to draft a set of by-laws for the Society. Drs. Raphael, Darby, and Drane, committee.

On motion of Dr. Bell, the order of proceedings was so far modified as to permit Dr. Chipley, Chairman of the Committee on Vital Statistics, to make his report. The report is one of great interest, the investigations were thorough, and the writer accompanied the report with a map, by which the relative sanitary conditions of the various counties were designated.

On motion of Dr. Evans, the report was received and referred to the committee on publication.

The Society adjourned to meet at 7½ o'clock, P. M., to hear the address of the President.

According to adjournment, the Society met, and devoted a short time to the business of admitting members, and the nomination of members for honorary membership. Dr. Chipley nominated Prof. E. Bartlett, of New York. Dr. Gross nominated Prof. Drake, of Ohio. And Dr. Yandell nominated Dr. Deaderick, of Tennessee, as honorary members, which were received for future action.

Prof. Miller offered the following resolution, to lie over until next day, for consideration:

*Resolved,* That the members will faithfully comply with the requisitions of the registration law passed at the last session of the Kentucky Legislature, and do what they can to have it complied with throughout the State.

Dr. Bell moved that Professor Caldwell be invited to meet the Society during its sittings, and participate in its deliberations. Passed unanimously.

The President, Dr. Sutton, then read the annual address, replete with matter of great interest, and with sound advice for elevating the character of the profession. On motion the address was referred to the committee on publication.

The Society adjourned until ten o'clock the next day.

SECOND DAY'S PROCEEDINGS.

Oct. 21—10 o'clock. The Society was called to order by the President. After the reading of the minutes, Dr. Gross moved the formation of the following special committees:

1. On Medical Biography, or the lives of meritorious or distinguished physicians or surgeons of Kentucky.

2. On Medical Literature, or the history of the medical authorship of Kentucky.

3. On the relation between diseases and peculiar geological formations.

4. On the Statistics of Hernia.

5. On the Statistics of Lithotomy and calculous diseases.

6. On the History and mode of management of Hospitals, Asylums, Infirmaries, Penitentiaries, and Prisons.

[This committee was afterwards divided, by assigning the two last named institutions to one committee, the others to another.]

7. On suits for malpractice.

8. On the results of surgical operations in malignant diseases.

9. On Epidemic Erysipelas.

10. On Epidemic Dysentery.

11. On Typhoid Fever.

12. On Placenta Previa.

And, at the suggestion of Dr. Bell, a Committee on the Statistics of Remedies.

Invitations were received from the Louisville University, the Kentucky School of Medicine, the Louisville Marine Hospital, and the Institution for the Blind, requesting the Society to visit those Institutions. All of which were received.

Professor Gross moved that Dr. Drake, of Cincinnati, be invited to take part in the deliberations of the Society.

Dr. D. D. Thompson, on behalf of the physicians of Louisville, invited the Society to a supper at the Louisville Hotel, on Friday night.

Prof. Flint moved that the election of officers should now be made, which was carried. After various ballotings, Dr. Chipley, of Lexington, was declared the choice of the Society for President. Drs. Bell and Drane were appointed a committee to conduct the President to the Chair.

Dr. Peter moved that a committee for nominating the remaining officers be appointed, consisting of one from each county, and one from the city of Louisville. The motion was carried, and the following gentlemen were appointed.

Dr. Darby, of Fayette; Dr. Bell, of Louisville; Dr. Owen, of Henry; Dr. Spillman, of Mercer; Dr. Sutton, of Scott; Dr. Chew of Woodford; Dr. Freeman, of Oldham; Dr. Foree, of Jefferson; Dr. Sneed, of Franklin; Dr. Letcher, of Jessamine.

Dr. Gross moved that the standing committee report in the order in which they stand in the proceedings of last year. Adopted; and on call, several of the committees failed to report. Dr. Evans, of Covington, read an able and well written report on Medical Ethics, which was received and referred to the committee on publication.

Adjourned to three o'clock, P. M.

AFTERNOON SESSION.

Dr. Chipley in the Chair. The society met pursuant to adjournment, and after the election of several members, the nominating committee reported candidates for the various offices; and upon a ballot for each office, the following officers were declared elected:

Dr. E. C. Drane, of Newcastle, Senior Vice President; Dr. J. W. Knight, of Louisville, Junior Vice President; Dr. Sneed, of Frankfort, Recording Secretary; Dr. R. J. Breckenridge, of Louisville, Corresponding Secretary; Dr. D. D. Thompson, of Louisville, Treasurer; Dr. B. Monroe, of Frankfort, Librarian; Drs. Bell, Foree, and Ronald, Committee of Publication.

Dr. Miller, chairman of the Committee on Obstetrics, then read a Report on Chloroform in Midwifery, and the use of the speculum in uterine diseases. The report commanded the profoundest attention; and that portion of it which examined the sentiments of Dr. Lee, of London, contained a triumphant refutation of that gentleman's opinions. The report was received and referred to the committee on publication.

Dr. Sutton read a report on Registration, which was read and referred to the committee on publication.

Dr. Jacobs offered a resolution respecting the clause in the constitution which restricts honorary membership to eminent physicians residing out of Kentucky. Ordered to lie over until the next day.

Adjourned to 7½ o'clock, P. M.

EVENING SESSION.

Dr. Chipley in the Chair. After the election of members, Dr. Wible offered the following preamble and resolution:

Whereas, Physicians are frequently called upon to give evidence in courts of justice, to make post mortem examinations, and institute investigations in cases of poisoning, services of a strictly professional character, requiring expense, time, and labor on the part of the physician;

And Whereas, these services are often of great importance to the welfare of society; and as physicians ought not to be expected to perform them without remuneration; it is the opinion of the Kentucky State Medical Society, that the interests of humanity demand that laws be enacted which will properly secure these services when required by coroners and other officers of the law;

*Therefore, be it Resolved,* That a committee of three be appointed to prepare a report, to be read at the next annual meeting of this Society, on the subject presented in the foregoing preamble.

[We may as well state that Dr. Sutton introduced a resolution for the appointment of a committee "to consider whether any, and if any, what means can be brought into requisition to lessen the heavy burthen of pauper practice, and report at the next annual meeting." The resolution and Dr. Wible's were referred to Dr. Wible, as chairman of the committee, with power to appoint coadjutors.]

Dr. Miller's resolutions, of yesterday, were adopted.

Dr. Darby, as chairman of the committee on Epidemics, read a report for himself, and one from Dr. B. P. Drake; and Dr. Allen, of the same committee, also read a report on Epidemic Dysentery.

The Society then selected Lexington as the place for the next annual meeting.

Adjourned to meet at 10 o'clock to-morrow.

THIRD DAY'S PROCEEDINGS—MORNING SESSION.

Dr. Chipley presiding, Dr. Gross's resolution for special committees was called up and adopted. Dr. Darby reported By-Laws, which report was suspended in order to hear Dr. Gross's report on Sur gery. This admirable report, one of the finest contributions that has been made to the medical literature of Kentucky, excited the deepest interest; and although expectations were very high, we believe the Professor transcended all anticipations. The report contains the most complete history of Kentucky surgery that has been written, and it would be considered a first class paper among the éloges of the French Institute The report was received and referred to the committee on publication.

Dr. Spillman made a remarkably excellent report on Indigenous Botany, which was referred to the committee on publication.

The following resolutions, appended to the report, were unanimously adopted:

*Rvsolved,* That this Society regard the cultivation of our own Botany as essential to a full development of our own professional resources, and to a more successful practice of our art.

*Resolved,* That the profession throughout the State, and the members of this Society particularly, be requested to give special attention to this subject, and by cultivating the field of observation and research, in connexion with the unexplored regions of vegetable nature within our own limits, ascertain to what extent the demands of the healing art can be supplied at home.

*Resolved,* That any physician discovering a new remedy, or a new property in one already known, or any information touching the Medical Botany of our State, that can be rendered practically available, be requested to communicate such information to the chairman of the committee on Indigenous Botany.

*Resolved,* That the committee of Publication, at as early a day as practicable, by a brief circular or otherwise, communicate the objects embraced in these resolutions to the profession throughout the State, giving the name and location of the chairman of the committee on Indigenous Botany, and earnestly requesting contributions.

Dr. Raphael's report on By-Laws was presented, received and adopted. Our limited space forbids the publication of these by-laws in the present number of the Journal.

Dr. Anderson offered a resolution of thanks to Col. Buckner for the use of the Court Room, and attentions to the Society. Unanimously adopted.

Various nominations for membership were acted upon, and the ballot was favorable in all instances.

The following appointments were announced by the President:

STANDING COMMITTEES.

| | |
|---|---|
| Committee of Arrangements...... | Dr. Darby, of Lexington. |
| " on Practical Medicine.. | Prof. Foree of Louisville. |
| " on Improvements in Pharmacy............... | Prof. Silliman, " |
| " on Vital Statistics...... | Dr. Sutton, of Georgetown. |
| " on Obstetrics.......... | Prof. Powell, of Louisville. |
| " on Medical Ethics...... | Dr. Hewett, " |
| " on Public Hygiene...... | Dr. Bell, " |
| " on Epidemics.......... | Prof. Bullitt, " |
| " on Surgery............ | Prof. Flint, " |
| " on Indigenous Botany... | Dr. Emmert, of Pike county. |
| " on Finance............ | Dr. Letcher, of Jessamine. |

SPECIAL COMMITTEES.

| | |
|---|---|
| 1. On Medical Biography.......... | Dr. R. J. Breckenridge, of Louisville. |
| 2. On Medical Literature, &c...... | Prof. L. P. Yandell, of Louisville. |
| 3. On Relations between Diseases and particular Geological Formations | Prof. Peter, of Lexington. |
| 4. On the Statistics of Hernia...... | Dr. S. B. Richardson, of Louisville. |
| 5. On the Statistics of Lithotomy and Calculous diseases............ | Prof. Gross, of Louisville. |
| 6. On the History and Management of Hospitals, &c............... | Dr. Raphael, " |
| 7. On Penitentiaries and Prisons.... | Dr. Sneed, of Frankfort. |
| 8. The results of Surgical Practice in malignant diseases............ | Dr. Colescott, of Louisville. |
| 9. On Epidemic Erysipelas........ | Dr. Owen, of Newcastle. |
| 10. On Epidemic Dysentery....... | Dr. Hynes, of Bardstown. |
| 11. On Typhoid Fever............ | Dr. C. H. Fry, of Louisville. |
| 12. Placenta Previa.............. | Prof. Miller, " |
| 13. On the Statistics of remedies in disease...................... | Prof. Rogers " |

Dr. Thompson, Chairman of Committee on Finance, made a report rom that committee. The report did not include the receipts at the recent meeting. Dr. Bell, Chairman of Committee on Case-Book, reported. He stated the extraordinary labors of Sir W. F. Chambers, of London. That gentleman has been engaged for many years in a most laborious private practice, in the management of a large hospital, and in teaching. He was also very sickly, yet he has kept a minute record of all the phenomena of all his cases, and has filled sixty-seven quarto volumes, each of 400 pages, with records of his cases. These records are all written in Latin. Sir William also made a number of Index books to his quarto volumes, and for years wrote up the hospital books in the style of his private case-books.

The committee urged the absolute necessity of a case-book for every practitioner, in which he may record the symptoms of every case. Two resolutions were appended to the report. The report was referred to the committee on publication, and the resolutions unanimously adopted. The following are the resolutions:

*Resolved,* That the Kentucky State Medical Society commend to the physicians of the State, and urge upon the members of the Society, the purchase of case-books of the kind described in this report.

*Resolved,* That in order that the publisher may determine how far he may be justified in putting this case-book to press, that the members present who may wish to encourage this publication, hand their names to the committee on publication, and that the committee shall be authorized to continue to receive these names after the adjournment to the next annual meeting, until a number shall be obtained sufficient to justify the enterprize; and when the committee shall have received this assurance, they shall be authorised to make arrangements with the publisher: Provided, the Society shall not be committed in any way for the expense of publication.

Dr. Raphael offered the following resolutions:

*Resolved,* That if the amount in the Treasury should not be sufficient to defray the expenses of getting out the proceedings of the Society, that the publishing committee assess the members *pro rata* with such excess.

*Resolved,* That any member may obtain one copy or additional copies, extra, of the transactions, by paying the cost price of the same.

*Resolved,* That the President be authorized to fill any vacancies in the delegation to the American Medical Association.

The following delegates were elected: Dr. Chipley, of Lexington, Dr. Freeman of Oldham; Dr. W. C. Sneed, of Frankfort; Dr. Raphael, of Lonisville; Dr. T. S. Bell, of Louisville; Dr. Gore, of Bloomfield; Dr. R. J. Breckenridge, of Louisville, Dr. Moore, of Harrodsburg; Drs. Wible, Hardin, and Hewett, of Louisville.

Prof. Bartlert, of New York; Prof. Drake, of Ohio; and Dr. Deadrick, of Tennessee, were then unanimously elected honorary members.

On motion of Dr. Jacobs, the thanks of the Society were tendered to the officers for the faithful, able, and impartial manner in which they had discharged their respective duties.

The Society then adjourned to meet at Lexington on the third Tuesday in October, 1853.

Thus closed the labors of this Society, and we sincerely believe that the meeting not only did a great service for the Medical profession, but that the good results will continue to show themselve for years to come.

We understand that the volume of Transactions will be put to press immediately; and it will be a book of which the medical men of Kentucky will have reason to be proud. We earnestly hope that every medical man in the State, who feels any interest in his profession, will order a copy of the work. The principal Reports will form a portion of the volume.

We understand, too, that the Committee of Publication are making arrangements with Mr. T. Nelson, of this city, for the immediate publication of the case-book referred to in the proceedings. It will be a proud day for medical science and practice in Kentucky, when all the practitioners of the State commence the use of the case-book. Those who begin it, will find such benefits to themselves and patients, that the practice will not be abandoned speedily. B.

